# Long-term outcomes of TEVAR for thoracic aortic diseases: a retrospective single-center study

**DOI:** 10.1186/s13019-024-02886-6

**Published:** 2024-06-29

**Authors:** Gokay Deniz, Ferit Kasımzade, Evren Ozcınar, Levent Yazicioglu, Sadik Eryılmaz

**Affiliations:** 1 Cardiovascular Department, Ankara Bilkent City Hospital, Bilkent Blvd. 1, Çankaya/Ankara, 06800 Turkey; 2 Cardiovascular Department, Ankara University, Bilkent Blvd. 1, Çankaya/Ankara, Turkey

**Keywords:** Endovascular repair, Surveillance, TEVAR, Thoracic aortic diseases

## Abstract

**Background:**

The outcomes of Thoracic Endovascular Aortic Repair (TEVAR) vary depending on thoracic aortic pathologies, comorbidities. This study presents our comprehensive endovascular experience, focusing on exploring the outcome in long term follow-up.

**Methods:**

From 2006 to 2018, we conducted TEVAR on 97 patients presenting with various aortic pathologies. This retrospective cohort study was designed primarily to assess graft durability and secondarily to evaluate mortality causes, complications, reinterventions, and the impact of comorbidities on survival using Kaplan-Meier and Cox regression analyses.

**Results:**

The most common indication was thoracic aortic aneurysm (*n* = 52). Ten patients had aortic arch variations and anomalies, and the bovine arch was observed in eight patients. Endoleaks were the main complications encountered, and 10 of 15 endoleaks were type I endoleaks. There were 18 reinterventions; the most of which was TEVAR (*n* = 5). The overall mortality was 20 patients, with TEVAR-related causes accounting for 12 of these deaths, including intracranial bleeding in three patients. Multivariant Cox regression revealed chronic renal diseases (OR = 11.73; 95% CI: 2.04–67.2; *p* = 0.006), previous cardiac operation (OR = 14.26; 95% CI: 1.59-127.36; *p* = 0.01), and chronic obstructive pulmonary diseases (OR = 7.82; 95% CI: 1.43–42.78; *p* = 0.001) to be independent risk factors for 10-year survival. There was no significant difference in the survival curves of the various aortic pathologies. In the follow-up period, two non-symptomatic intragraft thromboses and one graft infection were found.

**Conclusion:**

Comorbidities can increase the risk of TEVAR-related mortality without significantly impacting endoleak rates. TEVAR is effective for severe aortic pathologies, though long-term graft durability may be compromised by its thrombosis and infection.

## Introduction

Thoracic aortic diseases (TAD) represent a broad spectrum that includes thoracic aortic aneurism (TAA), aortic dissection (AD), penetrating aortic ulcer (PAU), intramural hematoma (IMH), traumatic aortic injury (TAI), and aortic coarctation (AC) [1]. Thoracic Endovascular Aortic Repair (TEVAR) has become increasingly favored for treating all Thoracic Aortic Dissections (TAD) due to its procedural simplicity and greater adaptability compared to open surgical repair [2, 3]. . Indications for TEVAR are expanding, new graft brands are being developed, and the success of endovascular operations is improving. Despite several advantages, there are still concerns about TEVAR’s durability, and surveillance is necessary to assess operational success in different aortic pathologies and the relationship with comorbidities [3, 4]. Endoleaks, such as type I, II, and III, require close monitoring due to the corresponding increased rupture risk. Graft breakage, graft defects, and stent migration are other concerns. Likewise, post-implantation syndrome, or graft thrombosis and intraluminal mural thrombosis cause for concern [5, 6]. Complication management and reintervention following TEVAR depend on clinical experience and perspective.

Therefore, studies on TEVAR, including long-term results, complications, and management strategies, continue to contribute to the literature [7, 8]. This retrospective study will share clinical TEVAR experiences and long-term follow-up results, while exploring risk factors in ten years.

## Materials and methods

### Data source and study design

**Fig. 1:**
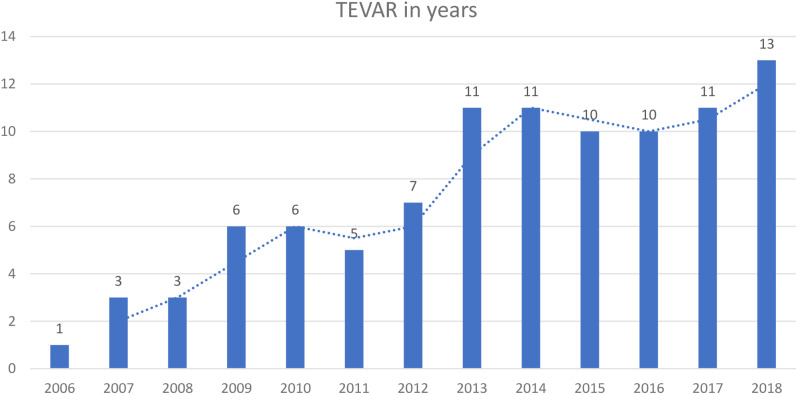
Numbers of TEVAR procedures over the years. The number of TEVAR procedures performed at the study clinic from 2006 to 2018

The data were retrospectively obtained from the hospital database at Ankara University, which includes patients who were treated with TEVAR from 2006 to 2018. Our first patient was a 37-year-old male with TAI due to a traffic accident, and it was one of the first TEVAR applications in Turkey. The number of procedures almost increased yearly, and 103 patients were treated over 12 years (Fig. 1). Six patients were excluded due to inadequate surveillance.

### Procedural details and strategies

TEVAR was performed for a broad spectrum of aortic pathologies in a hybrid operating room. Endografts were applied through the femoral arteries under general or local anesthesia. TAA was the most treated aortic disease, and AD followed. Only one patient treated with TEVAR had Stanford type-A AD. Indications for Stanford type B aortic dissections were aortic enlargement above 5.5 mm, persistent chest pain, or complicated dissection, as previously described in the literature [9, 10]. Hybrid operations were performed as needed. Before the TEVAR procedure, arch debranching surgery with Dacron tube grafts in sealing zone 0 and a left carotid-subclavian bypass (CSB) in sealing zone 2 were utilized to secure the proximal landing of endografts. Selective cerebrospinal fluid drainage (CFD) strategy was utilized for the prevention of spinal cord ischemia (SCI) at the high risk of spinal cord ischemia (SCI) as previously suggestions [11].

### Variables

We obtained demographic data, comorbid diseases, laboratory results, radiological images, and clinical and operational details from archive files or telephone clinical assessments. All enrolled patients were documented according to their indications and aortic pathologies. Preoperative risk stratification based on the American Society of Anesthesiologists physical status classifications (ASA) was reviewed [12]. The surgical procedure noted patients with CFD, CSB, and T8 segment coverage. Complications and adjunctive procedures were determined. Primary endoleaks detected in the operation room were addressed as needed. In the postoperative period, endoleak detection was performed using a contrast-enhanced CT scan. Aortic arch variants, anomalies, endoleak classification, measurements, graft landing zones, and graft landing lengths were investigated. Graft brand, diameter, length, and used number of grafts, and proximal landing zones (PLZ) based on Ishimaru’s classification [13] were explored. Complications were documented, long-term outcomes were investigated according to the PLZ. Hypertension, hyperlipidemia, chronic obstructive pulmonary disease, peripheral artery disease, cancer, diabetes mellitus, atrial fibrillation, heart failure, anticoagulant use, previous cardiac intervention, and chronic renal failure was considered comorbid diseases.

### Outcomes

The endograft durability in TEVAR over time was explored to assess the endograft patency and thrombosis, endoleak incidence, graft migration, reinterventions, and survival. The primary outcome explored in this study was graft durability over time. The secondary objective was to investigate the leading causes of mortality, complications, and reinterventions, evaluate the effects of comorbidities on procedural-related mortality and occurrence of type 1, 2, and 3 endoleaks. Long-term survival based on etiology and PLZ was also investigated.

### Statistics

All statistical analyses were performed using SPSS 20.0 for Windows (SPSS Inc, Chicago, IL). Significance was accepted below *p* < 0.05 in all groups. Confidence intervals (CIs) were set at 95%. While exploring the effects of graft diameter and sealing length on mortality and morbidity, graft characteristics were categorized using cut-off points identified through ROC (Receiver operating characteristic) analysis. Potential risk factors on 10-year survival and endoleak occurrence were investigated with Cox regression analysis. Kaplan-Meier analyses were performed according to aortic pathologies and PLZ.

## Results

During the follow-up period, a total of 103 patients underwent surgery due to TADs, with only six patients lacking surveillance data, resulting in a surveillance rate of 94%. The study evaluated 97 compliant patients between 2006 and 2018. The mean age of the patients was 61.6 ± 1.4 years, with the youngest patient being a 24-year-old with AC and the oldest patient being an 87-year-old with TAA. Seventy-two of the patients were male, constituting 74% of the cohort. Fifty-two had TAA, 29 had AD, six had TAI, four had IMH, five had PAU, and one had AC. Forty-two were classified as ASA 2, 35 as ASA 3, and 20 as ASA 4. Five had blunt thoracic injuries, one had an iatrogenic aortic injury, nine had ruptured TAA. A total of 15 TEVAR procedures were performed under emergency conditions. Aortic debranching surgery was performed for four patients to secure the landing zone, and TEVAR with zone 0 sealing was performed. The other landing zones were zone 2 sessions for 32 patients, zone 3 for 33 patients, and zone 4 and below graft landing for 28 patients. In the TEVAR procedure, a single graft was used for 69 patients, two grafts for 23 patients, and three grafts for five patients. In total, 131 TEVAR grafts were used. Medtronic Valiant® grafts were used in 73 patients, and the most used graft size was Medtronic Valiant® 46 × 46 × 200 (Table 1). The most common arch variation was a bovine arch found in eight patients, including the right-left brachiocephalic artery in one patient. The cohort had various comorbidities: 76 had hypertension, 43 had dyslipidemia, and 25 had chronic obstructive pulmonary disease and 21 had peripheral arterial disease. Fourteen patients had cancer, 11 had type 2 diabetes mellitus (none on insulin, all on oral antidiabetics), eight had atrial fibrillation, and eight had heart failure. A history of cardiac surgery was noted in 16 patients, including nine with CABG. Elevated creatinine levels were observed in 15 patients, with three requiring dialysis.

**Table 1:** Demographic data, intervention zones, detected arch anomalies, and graft brands

Age	Mean:61.64 Std: ±1.34 Min: 23 years-old Max: 87 years-old 95% CI: Lower Bound: 58.79 Upper Bound: 64.49			*N*	%
Male				72	74.23
**Etiology**					
	Thoracic Aortic Aneurism			52	53.61
		Type 1		34	35.05
		Type 2		10	10.31
		Type 3		3	3.09
		Type 5		5	5.15
	Thoracic Aortic Dissection				
		Type A^*^		1	1.03
		Type B		28	28.87
	Penetrated Aortic Ulcer			5	5.15
	Traumatic Aortic Injury			6	6.19
	Intramural Hematoma			4	4.12
	Aortic Coarctation			1	1.03
	Ruptured Aneurysm			9	9.28
		Due To Dissection		1	1.03
		Due To Aneurism		8	8.25
**Intervention Zone**					
	Zone 0			4	4.12
	Zone 2			32	32.99
	Zone 3			33	34.02
	Zone 4			28	28.87
**Aortic Arch Anomalies and Variants**				10	10.31
	Bovine Ark			8	8.25
	Commeral Diverticula			1	1.03
	Vertebral Artery Anomalies			1	1.03
**Graft Brands**					
	Gore Tag			20	20.62
	Medtronic			73	75.26
	Jotec Evita			4	4.12

The age and gender distribution of 97 TEVAR patients and the thoracic aortic diseases treated, categorized by etiology. The proximal aortic landing zones are classified according to the Ishimaru classification. It also lists aortic arch variations and anomalies detected in the treated patients. The brands of the grafts used are documented. *One patient with Type B aortic dissection subsequently developed retrograde aortic dissection, which progressed to Type A dissection

The secondary interventions, adjunctive procedures, and concomitant procedures were all documented (Table 2). The most common complication was endoleaks. In the 10-year follow-up, 15 patients had endoleaks including type 1a in six patients, type 1b in four patients, type 2 in five patients, type 3 in three patients, and type 5 in three patients. Some patients had multiple endoleaks. The treatment for type 1 endoleaks was TEVAR or balloon angioplasty. Glue or coil embolization was used for type 2 endoleaks. Two type 3 endoleaks following multiple graft applications were treated with balloon dilatation. One type 3 endoleak and all type 4 endoleaks disappeared in follow-up without intervention. No type 4 endoleak was observed.

**Table 2:** TEVAR complications and re-interventions

Complications										*n*	%
	Endoleak*									15	15.46
		Type I								10	10.31
				Type Ia						6	6.19
				Type Ib						4	4.12
		Type II								5	5.15
		Type III								3	3.09
		Type V								3	3.09
	Neurologic Complications									8	8.25
		Subdural Hematoma (SDH)								1	1.03
		Subarachnoid bleeding (SAB)								3	3.09
		Epidural hematoma								1	1.03
		Stroke								3	3.09
		Spinal Cord Ischemia (SCI)								3	3.09
	Contrast Nephropathies									2	2.06
	Groin Incision Complications									6	6.19
		Seroma								3	3.09
		Hematoma								2	2.06
		Peripheric Vascular Complications								1	1.03
	Graft Infection									1	1.03
	Upper Limp Ischemia									1	1.03
	Bowel Ischemia									1	1.03
	Retrograde Aortic dissection									1	1.03
	Vertebrobasilar Insufficiency									1	1.03
**Re-interventions**										**n**	**%**
Total re-interventions										18	18.56
TEVAR										5	5.15
Coil Embolization										3	3.09
EVAR										3	3.09
Bowel Resection										1	1.03
Iliac Artery-Mesentery Artery Bypass										1	1.03
Subdural Hematoma Drainage										2	2.06
Supraaortic Revascularization (SAR)										1	1.03
Carotis-subclavian Bypass (CSB)										1	1.03
Baloon Angioplasty										2	2.06
Femoral-femoral Artery Crossover Bypass										1	1.03
Stent to Subclavian Artery Aneurysm										1	1.03
Groin Revision										3	3.09
Carotid-carotid Artery Bypass										1	1.03

Complications encountered in the study cohort. Additional interventions deemed preoperative procedures, treatments of complications, or required interventions during follow-up are documented.* The total number of patients with observed endoleaks during follow-up is presented. Subsequently, the types of detected endoleaks are described. In some patients, multiple endoleaks were observed concurrently

**Fig. 2:**
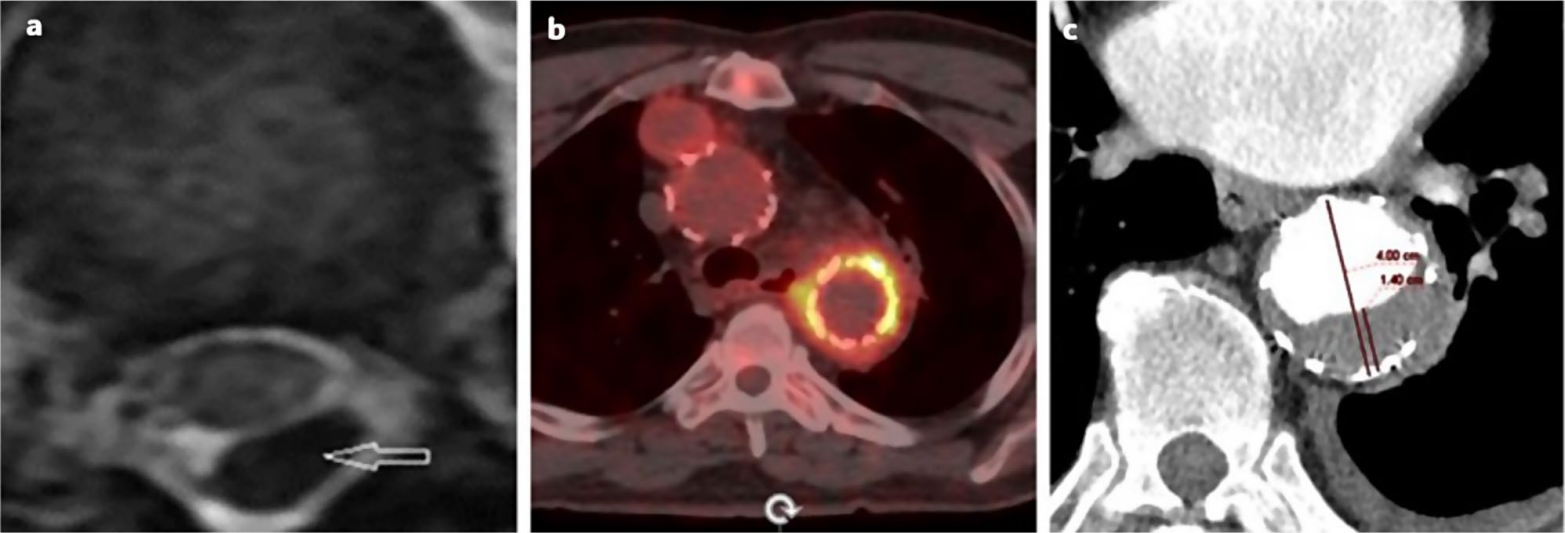
Rare complications in TEVAR procedures. Figure 2**a** (left): Epidural hematoma after cerebrospinal fluid drainage occurred in 44 years-old female on postoperative day five, Fig. 2**b** (middle): Graft infection was detected in 44 years-old male with PET CT. The patient who was treated with empiric broad-spectrum antibiotic therapy has recovered., Fig. 2**c** (right): 14 mm diameter asymptomatic intragraft thrombosis were detected in 64 years-old male on the 4th year-follow up

Neurological complications were observed in eight patients. Intracranial hemorrhage (ICH) occurred in four patients, including one subdural hematoma and three subarachnoid hemorrhages. Prophylactic CFD was selectively applied to 20 patients due to high SCI risk. CSB was performed in 22 of 33 patients with zone 2 landing. SCI and paraplegia occurred in three patients (3%) and cerebrovascular events occurred in two patients (2%) occurred. Permanent paraplegia and spinal cord ischemia in 2 patients occurred. An epidural hematoma that caused temporary paraplegia developed in one patient and was related to the spinal fluid drainage catheter (Fig. 2a). Access site complications were observed in five patients. One patient had a peripheric embolism, and an embolectomy was performed. Short-segment dissection in femoral access occurred, and the femoral artery was repaired in one patient. Contrast nephropathy was observed in 10 patients. Retrograde dissection was seen in one patient. TEVAR graft infection, a rare complication, occurred in one patient, who exhibited symptoms such as fever, weight loss, elevated inflammatory blood markers months after the treatment. Although the blood cultures were negative. Fluorodeoxyglucose positron emission tomography provided a conclusive diagnosis with uptake of fluorodeoxyglucose (Fig. 2b). Asymptomatic stenosis in the graft lumen was diagnosed in two patients postoperatively in the fourth and seventh years. Antiplatelet treatment was used for both, and no complications occurred in the follow-up (Fig. 2c).

In the follow up period, twenty patients died following TEVAR, and total mortality rate was 21%. Eight mortality reasons were classified as unrelated to the procedure, including causes such as cancer and chronic conditions. Excluding these eight mortalities, TEVAR-related mortality accounted for 12 deaths, with eight occurring within the first month due to complications such as ruptures and cardiac arrest (Table 3). Short-term mortalities (30-days mortality) were primarily due to acute procedural complications. The most common reason for TEVAR-related mortality was rupture (*n* = 4). Acute aortic syndromes, including AD, PAU, IMH, TAI, and ruptured TAA were found to affect 53 patients (54%). The effects of the comorbidities and factors over the occurrence of secondary type 1, type 2, and type 3 endoleaks after and procedure-related mortality at 10 years were investigated. Age did not increase operative mortality or morbidity (*p* = 0.62, 95% CI: 0.94–1.03). COPD increased TEVAR-related mortality (*p* = 0.018, 95% CI: 1.43–42.78). A history of cardiac operation also increased mortality (*p* = 0.01, 95% CI: 1.59-127.36). Furthermore, the mortality rate was higher in the chronic renal failure group (*p* = 0.006, 95% CI: 2.04–67.2). Mortality also increased as the length of graft sealing increased to 25 cm or longer (*p* = 0.08, %95 CI: 1.59–22.56). There was no relationship between the use of 38-mm-diameter endografts or larger and mortality. As the number of grafts used increased, mortality increased. Using three grafts or more was associated with significantly worse survival (*p* = 0,035). ASA classifications were correlated with TEVAR-related mortality. Patients with ASA 4 had significantly higher mortality (*p* = 0,006). No factors influencing the occurrence of type 1, 2, or 3 endoleaks were identified. In terms of the operational details, 25 cm and above graft sealing and T8 coverage were associated with increased endoleak risk; however, it was not significant (OR:3.35; 95% CI: 0.88–1.01, *p* = 0.052 and OR:3.24; 95% CI: 0.92–11.46; *p* = 0.06, respectively) (Table 4).

**Table 3:** Overall causes of all mortality

						*N*		%
**Causes of All Mortality After TEVAR**						20		20.62
**Causes of Procedure-Related Mortality**						12		12.37
		Rupture				3		3.09
		Rupture after implantation				1		1.03
		Cerebrovascular events				3		3.09
		Cardiac arrest during the procedure				1		1.03
		Sepsis				1		1.03
		Bowel Ischemia				1		1.03
		Peripheral arterial embolism				1		1.03
		Aortobronchial fistula				1		1.03
**Other Causes**						8		8.25
		Cancer				3		3.09
		Crush Syndrome				1		1.03
		Sepsis				1		1.03
		Cardiac				1		1.03
		Pneumonia				1		1.03
		EVAR related				1		1.03

Categorization of all causes of mortality observed after TEVAR treatment classified as procedure-related causes or other causes

**Table 4:** Cox regression analysis outcomes for 10-year TEVAR-related mortality and comorbidity risk estimation for endoleak occurrence

	*n*	Mortality	CI	OR	*P*			Endoleak	CI	OR		*p*	
Demographical Characteristics													
Age			0.94–1.03	0.98		0.62			0.95–1.03		0.99		0.86
Sex (Male)	72	11	0.02–1.79	0.216		0.62	13		0.12–2.82		0.58		0.5
Comorbidities													
Hypertension (HT)	76	9	0.12–7.23	0.94		0.95	14		0.50-33.36		4.11		0.18
Hyperlipidemia (HL)	43	3	0.02–0.92	0.15		0.04	9		0.26–3.79		0.99		0.99
Chronic Obstructive Pulmonary Disease (COPD)	25	8	1.43–42.78	7.82		0.01	5		0.73–9.21		2.6		0.13
Peripheral Arterial Diseases	21	3	0.09–3.38	0.55		0.52	3		0.22–6.79		1.23		0.81
Cancer	14	3	0.19–7.70	1.23		0.82	2		0.61–20.45		3.54		3.54
Diabetes Mellitus (DM)	11	2	0.44–64.69	5.37		0.18	1		0,16-17.95		1.71		0.52
Atrial Fibrilation (AF)	8	2	0.33–48.97	4.06		0.27	1		0.35-116.67		6.44		0.2
Chronic Heart Failure (CHF)	8	1	0.06–23.93	1.21		0.89	0		0-.		0		0.98
Anticoagulant Usage	11	1	0.01–1.59	0.11		0.1	3		0.23–13.4		1.78		0.57
Previous Cardiac Operation	16	4	1.59–127.3	14.26		0.01	5		0.2–9.55		1.38		0.74
Chronic Renal Disease (CRD)	18	5	2.04–67.20	11.73		0.006	1		0.03–2.82		0.3		0.65
ASA score													
2	42	3	-	-		0.01	5		-		-		0.79
3	35	3	0.24–6.09	1.23		0.8	9		0.58–5.24		1.75		0.31
4	20	6	1.89–48.16	9.55		0.006	1		0.17–13.31		1.52		0.7
Operational comorbidity													
T8 cover	48	8	0,53 − 6,69	1,8		0,32	11		0,88 − 1,01		3,35		0,052
Carotid-Subclavian Bypass (CSB)	22	4	0,36 − 4,05	1,2		0,75	6		0,18 − 2,12		1,61		0,44
Cerebrospinal Fluid Drainage (CFD)	20	4	0,45 − 5,81	1,6		0,45	4		0,19 − 1,51		1,8		0,26
Comorbidities related to Endograft Features													
38 mm and above	38	7	0,28 − 4,03	1,08		0,18	7		0,36-,4,29		1,25		0,72
25 cm and above	20	7	1,59 − 22,56	5,99		0,08	6		0,92 − 11,46		3,24		0,06
The number of endograft													
1	74	5	-	-		0,59	11		-		-		0,39
2	18	4	0,85 − 10,72	3,02		0,87	2		0,20 − 4,23		0,92		0,92
3	5	3	1,13–27,93	5,62		0,035	2		0,61 − 12,62		2,78		0,18

The impact of predicted comorbid factors based on demographic, operational, and endograft characteristics, on procedure-related mortality. Subsequently, the effect of these factors on the development of type 1, 2, and 3 endoleaks has been investigated

**Fig. 3:**
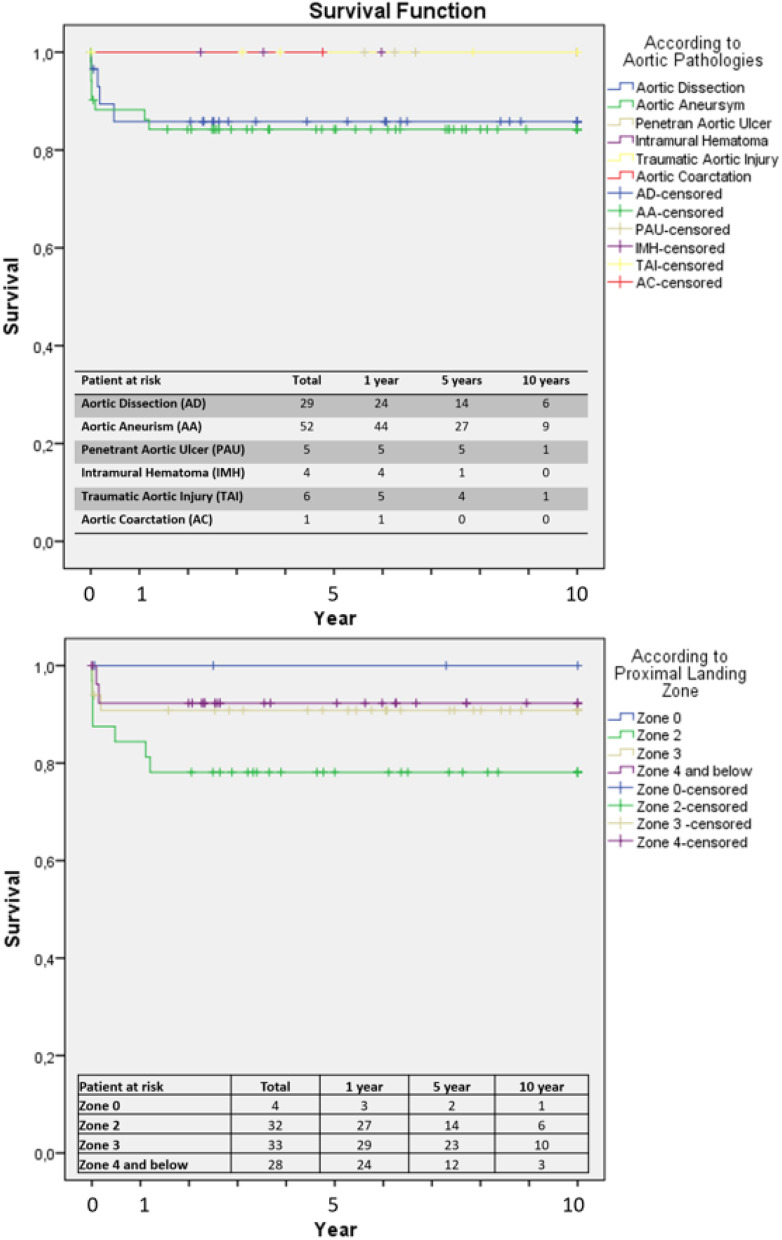
Kaplan-Meier long-term survival curve comparing etiology and intervention zone regarding Ishimaru classification. Visual representation of survival probabilities over time for providing sights into the effectiveness of interventions based on etiology and intervention zone

There was no significant difference beyond etiology-based long-term results in the Kaplan-Meier survival curves regarding TEVAR-related mortality (*p* = 0.35). Cumulative TEVAR-related mortality was 8% for one month, 9% for six months, 10% for one year, 13% for five years, and 13% for ten years. According to aortic pathologies, the highest mortality rate was observed in ruptured aortic aneurysms in the survival analysis. Mortality and secondary type 1, 2, and 3 endoleaks were undetected in TAI, PAU, and AC. Survival analysis between groups was performed using Kaplan-Meier analysis, and there was no significant difference in the log-rank test regarding aortic pathologies and proximal landing zones (*p* = 0.76, *p* = 0.27). Survival curves for AD and TAA were similar. There was no mortality at zone 0 TEVAR landing following aortic debranching surgery. The highest mortality rate was observed in the zone 2 landing. Although zone 4 interventions had relatively more minor mortality, this was not statistically significant (*p* = 0.27) (Fig. 3).

## Discussion

TADs are one of the main interests of cardiovascular surgeons due to their important mortality and morbidity rates. With the widespread usage of diagnostic tests, the number of patients diagnosed with TADs has increased. TEVAR is increasingly being applied because of its low cost, easy application, and adaptability [3]. This study shows a similar increase in patients who underwent TEVAR over the years. Additionally, hybrid interventions for complicated aortic diseases have increased [14]. In this study, aortic debranching surgery was performed for four patients with zone 0 landings, however there was no zone 1 landing TEVAR. The efficacy and outcomes of TEVAR differ depending on treated etiology and relevant comorbidities. Long-term research and improved surveillance are still crucial for assessing endograft durability.

Upon retrospective searching, arch anomalies and variants were detected in 10 patients, and the most common arch variant was a bovine arch. The incidence rates of aortic variants align closely with those found in the literature [15]. Variants are more prevalent in patients with AD due to flow hemodynamics [16]. Nonetheless, there was no difference between the aortic pathologies. TEVAR treatment is safe and effective in arch anomalies and variants, even if the procedure seems complicated.

TEVAR-related complications remain the biggest problem for the procedure. Recipients should be closely followed for complications. This is especially true of endoleaks which have a high incidence (4–24%) [17]. Endoleaks were observed at a rate of 15% in this series. Some types of endoleaks can lead to other types. In one case, a type 2 endoleak was encountered because the left subclavian artery led to a type I endoleak involving retrograde dissection. The incidence and mortality of retrograde aortic dissection are 2.5% and 37%, respectively [15]. In this case, two times repeated TEVAR interventions were performed. No endoleak or dissection was detected in the follow-up after the last intervention. Therefore, early detection is crucial to avoid complicating treatment. Type 1 endoleaks are the most common endoleaks in our series and the type most requiring intervention. Balloon dilatation was the first solution for primary and secondary type 1 endoleaks. However, repeated TEVAR was applied in some cases. Type 2 endoleaks were the second most common type of endoleak. They mainly occurred because the left subclavian artery with zone 2 landing TEVAR created the need for reintervention. Coil embolization could be a good solution for solving type 2 endoleaks. Type 3 endoleaks were observed in accompanying multiple graft usage, and this was solved with balloon dilatation. Type 5 endoleaks were found in three patients with aneurysm sac enlargement without any detected leakage. Each case was followed, and the resolution of all type 5 endoleaks was maintained without any intervention. There were no type 4 endoleaks.

Neurological complications related to TEVAR are the most dreaded complications due to related with high mortality, even though they are rare. The incidence of paraplegia after TEVAR can range from 0 to 12.5%, but it is commonly between 3% and 6% [18]. CFD’s proven ability to lower SCI rates in open thoracic aortic surgery by increasing spinal cord perfusion pressure, has made it a preferred method for SCI prevention during TEVAR. The ongoing debate over CFD usage in TEVAR continues due to insufficient evidence whether it reduces the incidence of SCI sufficiently to justify the additional risks involved [19]. Some surgeons perform prophylactic CFD in all patients undergoing TEVAR, while others perform selective CFD using salvage CFD only when necessary. It has been reported in a historical paper that 8% of paraplegia is seen in TEVARs performed without CFD. A systematic review showed that the pooled SCI rate without routine prophylactic drainage was around 1.98–5.37%, even though the SCI rate with regular prophylactic drainage being 1.7–5.1% [20]. In addition, CFD can cause some complications such as infection, epidural hematoma, subdural hematoma, intracranial bleeding that are closely related to CFD [21]. In this study, preoperative selective CFD was performed in patients with high SCI risk; however, paraplegia still occurred in three patients. There was no clear evidence that CFD decreased the risk of SCI, whereas ICH was high in this series and may be linked with CFD. ICH occurred in three patients, resulting in death, and could be associated with CFD. Subarachnoid hemorrhage occurred in two patients, and subdural hematoma occurred due to extensive drainage during the treatment of SCI following TEVAR in one patient. Epidural hematoma is a rare complication of CFD [22]. In one case, after unilateral paralysis developed on the second postoperative day, the patient was diagnosed with epidural hematoma (Fig. 2a). The paraplegia regressed in the follow-up and healed without any sequelae. The therapeutic window of CFD can be narrow for both treatment and prevention of SCI. Stroke is another serious neurological complication associated with high mortality after TEVAR. In the literature, the stroke rate is between 2% and 8% [23]. In this study, stroke occurred in three patients.

The rate of bowel ischemia is only around 0.6-2.8% in TEVAR; but, it has highly mortality rate [24]. In one patient in this study with complicated type B dissection, the false lumen closed, and mesenteric ischemia developed with the expansion of the true lumen. Abdominal pain and an elevated lactate value indicated bowel ischemia with an inadequate collateral network. The diagnosis was confirmed with emergency laparotomy. Although the patient underwent an iliac artery mesenteric superior bypass, the complication resulted in death due to reperfusion injury. Some patients with inadequate collaterals can have an increased risk of mesenteric or renal ischemia following the closure of the false lumen in TEVAR treatment for AD when the dissection segment lays down to the abdominal aorta. Surgical treatment could be a better solution in this case.

Intragraft thrombosis has been reported in minimal case studies as a late device-related complication [25]. Two cases of intragraft thrombosis were detected in this study, both in patients with less than 50% stenosis and no symptoms (Fig. 2c). There was no need for additional intervention because of the asymptomatic prognosis. We continued treatment with antiplatelet therapy. There was no embolic complication in the follow-up.

In this study, 74% of the patients who underwent TEVAR were male, and 26% were female, with a ratio of men to women of 3:1. We found that gender did not affect the outcomes as like the literature [26]. There was a wide range in age disturbance. Previous research has shown that endovascular repairs yield similar outcomes in young and elderly patients [27]. Despite lacking enough evidence on long-term durability in younger patients, some medical centers preferred TEVAR in adolescents [28]. Age was not associated with higher odds of mortality and endoleak in this study. A 23-year-old patient with TEVAR and an 87-year-old patient with TAA, the youngest and the oldest patient in our series, had a successful mid-term result. The relationship between comorbid factors and TEVAR mortality, procedural success, and endoleak has been investigated in many studies [1, 29–31]. As in the literature, this study found that chronic obstructive pulmonary disease and chronic renal disease increased mortality [32]. Additionally, our findings indicate a correlation between higher ASA scores and increased mortality rates. Preoperative ASA status can predict perioperative outcomes [33]. Risk factor analysis through receiver operating characteristics curves identified graft characteristics, notably that mortality rates increased with aortic coverage exceeding 25 cm. While endoleak and mortality rates were higher in grafts exceeding 38 mm in diameter, the association did not reach statistical significance. As the number of grafts increased, the success of the procedure decreased. Longer or custom-made grafts could improve these outcomes.

Long term survival curves showed no significant differences according to etiologies and landing zones. No mortality or endoleaks were seen in PAU, AC, or TAI. TEVAR procedural success for these TADs has also been high in other series [32, 34]. Significantly, the results of TEVAR for TAI was considerably superior [34, 35]. The observed association of Ishimaru zone with mortality increases as the landing zone goes proximally [36]. Although there was no significant association due to the small sample size, zone 2 and zone 3 sealing had higher mortality rates. The results in the zone 0 landings encouraged us to use hybrid endovascular interventions [37]. TEVAR can offer preferable solutions to all TADs in any intervention zone.

The research has some limitations because it is a single-center retrospective study with a small sample size. The limited number of patients and the heterogeneity of the intervention zone, etiologies, make statistical inferences challenging. Despite their small numbers, the literature needs long-term single-center studies with comprehensive explorations of TEVAR treatments.

## Conclusion

Multicenter trials have confirmed that TEVAR can be used safely to treat TAD. However, rare complications still limit the outcomes. Hybrid interventions can offer a tailored treatment strategy for complex diseases. With long-term results, TEVAR can provide successful treatment suitable younger and older patients. Patients with high ASA status, chronic obstructive pulmonary disease, and chronic renal diseases had increased risk of procedure-related mortality. Using endografts that exceed 25 cm in length, or 38 mm in diameter, or using more than two endografts may elevate the associated operational risk. Close monitoring and adherence to the surveillance protocols for these patients is recommended.

## Data Availability

The datasets generated and analyzed during the current study are available from the corresponding author on reasonable request.
